# Improving the Accuracy of ED Triage Using the Australasian Triage Scale: A Closed-Loop Quality Improvement Project

**DOI:** 10.7759/cureus.106424

**Published:** 2026-04-04

**Authors:** Mohamed Ahmed Abugibba Mohamed, Ahmed Mohammed Abdullah Abdulaziz, Mohammed Hasan Ahmed Al-Attas, Khalid Mustafa Yousif Osman, Mohammed Fadhl Salem Dahloos, Abdullah Abdulsadeq Salem Bawazir, Mustafa Hassan Ali Mohamed, Sanad Ahmed Abdulrahman Al-Mansori, Mohammed Saleh Mohammed Basleem, Mohammed Hussein Mohammed Muwadh, Musab Okasha Mohamed Ibrahim, Mahmoud Abdelrazik Abdellatif Elmewafy, Weam Habib Alla Mohamed Nour, Hebatallah Ali Abdeen Mohamed

**Affiliations:** 1 Internal Medicine, 6 October Hospital, Dokki, EGY; 2 Emergency Medicine, 6 October Hospital, Dokki, EGY; 3 Internal Medicine, Al Neelain University, Khartoum, SDN

**Keywords:** australasian triage scale, clinical audit, emergency department, patient safety, quality improvement, triage

## Abstract

Background and aim

Accurate ED triage is essential for patient safety, timely care, and effective resource utilization. The Australasian Triage Scale (ATS) provides a standardized framework for prioritizing patients based on clinical urgency; however, variability in its application may compromise care quality. This clinical audit aimed to assess compliance with the ATS in an ED and to evaluate the impact of targeted quality improvement interventions on triage accuracy.

Methods

A prospective closed-loop clinical audit was conducted in the ED of 6 October Hospital, Dokki. A total of 868 triage encounters were reviewed across two audit cycles (434 cases per cycle). Baseline data were collected between October 1 and 15, 2025, followed by a three-week intervention consisting of structured education, bedside coaching, and cognitive support tools. Reaudit data were collected between November 8 and 22, 2025. The primary outcome was the proportion of correctly assigned ATS categories, and differences between cycles were analyzed using the chi-square test.

Results

At baseline, correct ATS category assignment was documented in 320 of 434 cases (73.8%), below the predefined audit standard of ≥90%. Following the intervention, correct triage assignment increased to 393 of 434 cases (90.6%), meeting the audit standard. This improvement in triage accuracy was statistically significant (χ² test, p < 0.001).

Conclusions

This audit demonstrated that targeted educational and system-based interventions significantly improved compliance with the ATS. The findings highlight the effectiveness of structured training, bedside coaching, and cognitive support tools in enhancing triage accuracy in high-volume emergency departments. Improved triage performance has important implications for patient safety, timely clinical decision-making, and optimal resource utilization. A closed-loop clinical audit represents a practical and sustainable approach for monitoring and improving triage practices, and similar strategies may be applied in other emergency care settings to strengthen the quality and consistency of care.

## Introduction

ED triage is a fundamental process in acute healthcare that prioritizes patients based on the urgency of their clinical condition. The primary aim of triage is to ensure that patients with the most critical needs are identified promptly and receive timely interventions, while those with lower acuity are managed appropriately within available resources. This prioritization is essential for patient safety, efficient workflow, and equitable access to emergency care in environments with high patient volume and limited resources [1].

Various standardized multilevel triage scales have been developed to support clinical staff in making consistent and evidence-based triage decisions. One widely used system in Australia and several other settings is the Australasian Triage Scale (ATS), a five-level urgency classification framework that links presenting complaints with maximum acceptable waiting times for medical assessment and treatment [2]. The ATS assists emergency nurses in quickly categorizing patients from Category 1 (immediate, life-threatening) to Category 5 (nonurgent), thereby facilitating appropriate prioritization and efficient resource allocation within the ED [3].

Despite the availability of structured tools such as the ATS, variability in triage performance remains a consistent challenge in ED settings. Research suggests that the reliability and accuracy of ATS assignments can be influenced by clinicians’ training, experience, cognitive load, and interpretation of triage criteria. A meta-analysis on the reliability of the ATS reported acceptable overall agreement but identified ongoing variability that may contribute to errors such as under-triage, which can delay care for critically ill patients [4]. Additional evidence from contemporary emergency nursing research demonstrates that triage accuracy rates vary considerably across institutions and are significantly influenced by nurse experience, workload intensity, and environmental pressures, further underscoring the need for structured quality improvement and ongoing performance monitoring [5]. However, much of the existing evidence originates from well-resourced EDs, and relatively limited data are available regarding ATS performance and variability in different healthcare contexts, particularly in busy departments with constrained resources. This highlights the need for local evaluations to better understand triage accuracy and identify opportunities for targeted quality improvement.

Inaccurate triage decisions have significant implications for patient outcomes and healthcare quality. Under-triage may lead to delayed critical interventions, increased morbidity and mortality, and prolonged ED length of stay, while over-triage can misallocate scarce resources and contribute to overcrowding. Emerging evidence emphasizes the need for continuous quality assurance mechanisms, including structured education, regular audits, feedback, and performance monitoring, to enhance triage accuracy and consistency [6].

A clinical audit represents a systematic method for evaluating current practice against defined standards, identifying gaps, and implementing targeted improvements. A closed-loop clinical audit involves completing the full audit cycle: measuring baseline performance, implementing interventions to address identified deficiencies, and conducting a reaudit to determine whether improvements have been achieved. In the context of ED triage, audit and reaudit cycles can provide valuable insights into compliance with triage guidelines such as the ATS, measure the effectiveness of interventions, and support the embedding of best practices into routine care. Regular evaluation of triage performance is therefore essential for improving patient safety, optimizing emergency care delivery, and ensuring alignment with evidence-based standards [7].

In the setting of a busy ED at 6 October Hospital, concerns regarding variability in ATS application and potential deviations from established triage standards prompted the need for systematic evaluation. The general aim of this clinical audit was to assess compliance with the ATS and the accuracy of triage category assignment in the ED. The specific objectives were to determine the proportion of patients correctly triaged according to ATS criteria during the baseline audit cycle, identify patterns and sources of triage misclassification, implement targeted educational and system-based interventions to address identified gaps, and evaluate the impact of these interventions through a structured reaudit cycle. By aligning local practice with internationally recognized triage standards, this audit sought to improve patient safety, optimize emergency care delivery, and support sustainable quality improvement within the department.

## Materials and methods

Study design

This study was conducted as a prospective, closed-loop clinical audit designed to assess compliance with the ATS and to evaluate the impact of targeted quality improvement interventions on triage accuracy within the ED. The audit followed a two-cycle structure consisting of a baseline assessment, implementation of corrective interventions, and a reaudit to measure improvement against predefined standards.

Study setting

The audit was carried out in the ED of 6 October Hospital, Dokki, a high-volume acute care facility providing emergency services to a diverse urban population. The department operates continuous triage services staffed by trained emergency nurses who assign ATS categories at initial patient presentation.

Audit standard

The audit standard was derived from the ATS guidelines, which recommend accurate assignment of triage categories based on clinical urgency and presenting features [2]. A performance threshold of ≥90% correct ATS category assignment was defined as the audit standard, in line with accepted benchmarks for triage accuracy reported in the literature and international emergency care practices.

Study population and sample size

All patients presenting to the ED during the defined audit periods and undergoing formal triage assessment were eligible for inclusion. The sample size of 434 cases per audit cycle reflects the total number of eligible triage encounters recorded during each two-week audit period, allowing comprehensive evaluation of routine triage practice during the study time frame. In total, 868 triage encounters were reviewed across both cycles. No cases were excluded due to incomplete or missing data, as all triage encounters had complete documentation required for ATS categorization.

Audit cycles and data collection

The baseline audit cycle was conducted over a two-week period from October 1 to 15, 2025, during which triage records were prospectively reviewed to assess the accuracy of ATS category assignment. The intervention phase took place over three weeks from October 16 to November 7, 2025, and involved the implementation of targeted educational and system-based measures. The reaudit cycle was subsequently conducted over a two-week period from November 8 to 22, 2025, using identical inclusion criteria and data collection methods to ensure comparability.

Data were collected using a standardized audit pro forma that recorded patient presenting complaints, assigned ATS category, and the expected ATS category based on guideline criteria. The expected triage category was determined through structured review against ATS definitions and descriptors, allowing classification of each case as either correctly or incorrectly triaged. To minimize potential assessment bias, case evaluations were performed using predefined ATS criteria and standardized reference materials.

Intervention

Based on gaps identified during the baseline audit, a multifaceted improvement package was implemented. This included formal educational sessions delivered through departmental lectures, bedside teaching and coaching during clinical shifts, distribution of ATS pocket reference cards, display of visual ATS posters within triage areas, and a structured online educational meeting. These interventions were designed to reinforce correct application of ATS criteria, improve clinical judgment, and promote consistent triage decision-making.

Outcome measures

The primary outcome measure was the proportion of patients correctly assigned an ATS category in each audit cycle. Secondary outcomes included the proportion of incorrectly triaged cases and overall compliance with the predefined ≥90% performance standard. Improvement was assessed by comparing triage accuracy between the baseline and reaudit cycles.

Data analysis

Data were entered into a secure database and analyzed using descriptive and inferential statistics. Categorical variables were summarized as frequencies and percentages. Differences in triage accuracy between audit cycles were assessed using the chi-square (χ²) test, with statistical significance set at p < 0.05. Results were presented to demonstrate changes in compliance with ATS standards following the intervention.

Ethical and governance considerations

This project was conducted as a clinical audit and quality improvement initiative and did not involve any alteration to standard patient care. All data were anonymized prior to analysis, and patient confidentiality was maintained throughout the audit process. Formal ethical approval was not required in accordance with institutional policies for service evaluation and clinical audit activities.

## Results

A total of 868 ED triage encounters were audited across two audit cycles, with 434 cases reviewed in each cycle. All included cases had complete triage documentation and were suitable for assessment against ATS criteria; no cases were excluded due to missing or incomplete data.

During the baseline audit cycle conducted between October 1 and 15, 2025, correct assignment of the ATS category was documented in 320 of 434 cases (73.8%), while 114 cases (26.2%) were incorrectly triaged. This level of performance fell below the predefined audit standard of ≥90% correct ATS assignment, indicating suboptimal compliance with ATS guidelines at baseline.

Following implementation of the targeted educational and system-based interventions, the reaudit cycle conducted between November 8 and 22, 2025, demonstrated a marked improvement in triage accuracy. Correct ATS category assignment was achieved in 393 of 434 cases (90.6%), while incorrect assignment was reduced to 41 cases (9.4%), thereby meeting and exceeding the audit standard.

Comparison of triage performance between the two audit cycles demonstrated a statistically significant improvement in ATS compliance following the intervention. The increase in correct triage assignment from 73.8% in Cycle 1 to 90.6% in Cycle 2 represented an absolute improvement of 16.8 percentage points. Chi-square analysis confirmed that this improvement was statistically significant, χ²(1, N = 868) = 38.62, p < 0.001. The effect size was also calculated (Cramér’s V = 0.21), indicating a small-to-moderate magnitude of association between audit cycle and triage accuracy, suggesting that the intervention had a meaningful practical impact.

**Figure 1:**
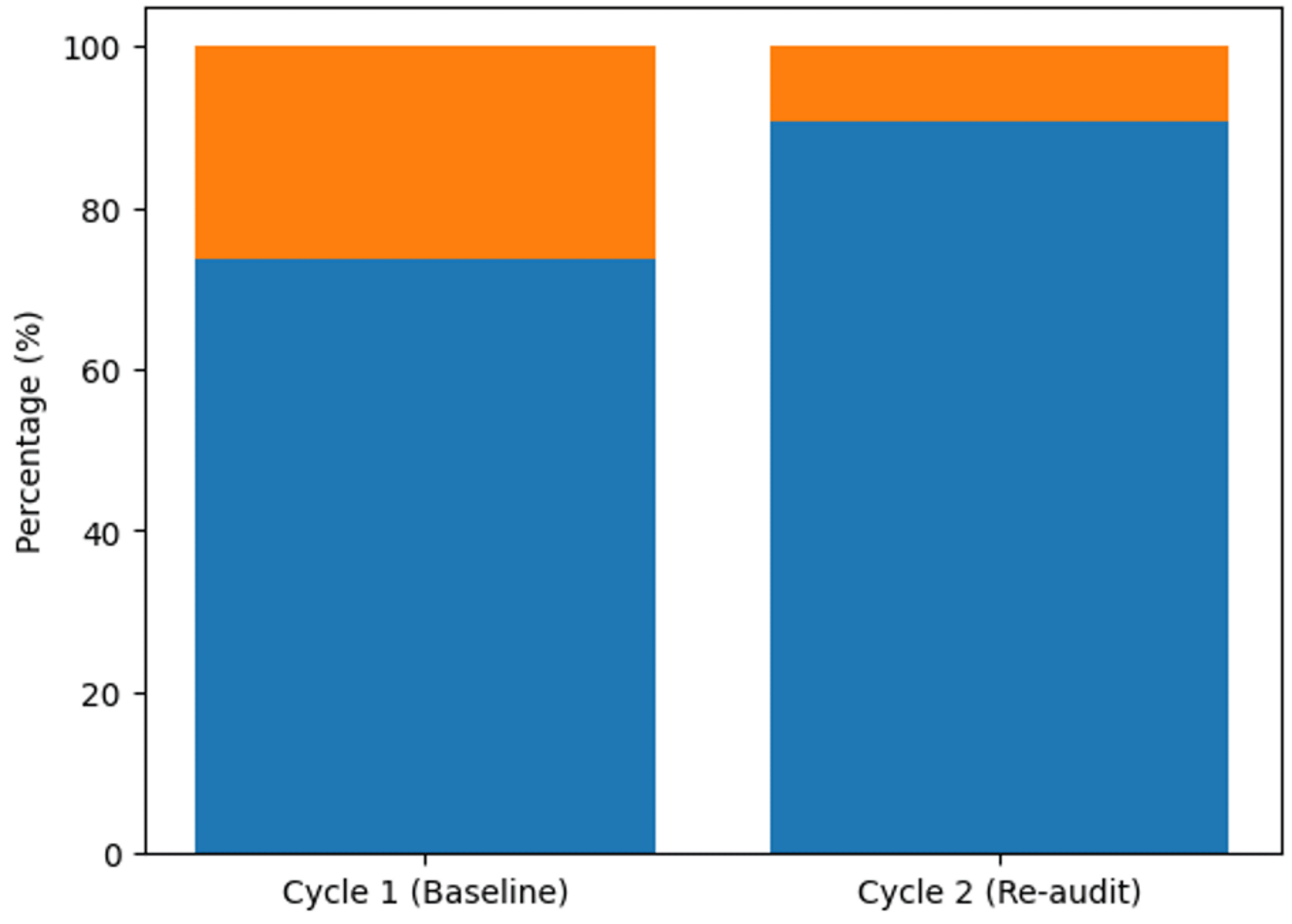
Accuracy of ATS category assignment before and after the quality improvement intervention This figure illustrates the proportion of correctly and incorrectly assigned ATS categories before and after the intervention, demonstrating a statistically significant improvement in triage accuracy. ATS, Australasian Triage Scale

Overall, the results indicate that the implementation of structured education, bedside coaching, and cognitive aids was associated with a substantial and statistically significant enhancement in the accuracy of ATS category assignment. The reduction in mis-triage following the intervention reflects improved adherence to triage guidelines and more consistent prioritization of patients according to clinical urgency (Figure 1).

## Discussion

This clinical audit demonstrated a substantial improvement in triage assignment accuracy following the implementation of structured quality improvement interventions. The increase in correct ATS category assignment from 73.8% at baseline to 90.6% after intervention confirms that targeted education and system enhancements can significantly enhance triage performance in high-volume EDs. This aligns with broader evidence indicating that structured audit feedback and educational initiatives are effective strategies for improving clinical practice standards [8].

Accurate triage is a critical component of emergency care quality because it directly influences patient prioritization and subsequent clinical outcomes. Evidence shows that nurses’ accuracy in triage assignment can vary significantly, with studies reporting wide-ranging rates influenced by clinician experience, training, and contextual factors. For example, triage accuracy has been observed to range between approximately 59% and 82% in various settings, highlighting persistent challenges in achieving consistent performance without targeted improvement strategies [9].

The consequences of triage misclassification extend beyond audit metrics and impact real patient outcomes. Under-triage, in particular, has been linked to delays in critical diagnostic and therapeutic interventions, with documented associations between under-triage and extended time to imaging, delayed medications, and prolonged length of stay for high-risk conditions such as subarachnoid hemorrhage and aortic dissection. These findings emphasize the safety imperative of accurate triage in EDs [10].

The current findings complement earlier work showing that multifaceted interventions, including education, audit feedback, visual aids, and structured coaching, are more effective than singular approaches. Comprehensive strategies are recommended as they address multiple determinants of clinical behavior, including knowledge gaps, cognitive processes, and organizational support systems. Systematic reviews suggest that continuous training, active nurse involvement, and validated audit tools are essential components of successful triage quality improvement programs [8].

One mechanism by which education and feedback improve triage performance appears to be through enhancing clinical reasoning and interpretation of triage criteria. Realist and behavioral science literature contend that interventions grounded in behavior change frameworks can influence both capability and motivation components of triage clinician behavior, potentially leading to more accurate and consistent decision-making in dynamic clinical environments [8]. Educational and structured training interventions have also led to measurable gains in triage competency and accuracy among ED nurses in multicenter pre-post studies, further supporting the effectiveness of targeted educational reinforcement strategies [11].

Comparison of our findings with other triage audit projects further validates the utility of this approach. A peer audit study reported an increase in appropriate emergency categorization from 63% at baseline to 90% at reaudit following targeted interventions, mirroring the magnitude of improvement seen in our audit and reinforcing the generalizability of structured quality improvement cycles in diverse clinical settings [12].

In addition to educational strategies, emerging evidence points toward the potential role of technology and decision support tools in augmenting triage accuracy. Innovative approaches, including game-based educational apps and electronic decision support systems, have shown promising results for engaging clinicians and facilitating rapid, contextually appropriate triage decisions. While these technologies are not yet standard practice, they may represent valuable adjuncts to traditional quality improvement efforts in future audits [8].

The literature also highlights the importance of understanding local contextual factors that influence triage performance. Characteristics such as patient population complexity, triage staff experience, resource availability, and departmental culture can moderate how interventions translate into practice. Cross-sectional studies assessing triage knowledge and practices reveal that even high levels of knowledge do not automatically translate into accurate triage decisions, pointing to the need for ongoing reinforcement and evaluation mechanisms [13].

Importantly, while this audit demonstrated significant short-term improvement, sustainability of gains over longer periods remains a priority. Evidence underscores the need for continuous monitoring, regular reaudit cycles, and embedding triage quality indicators into routine performance dashboards to prevent regression and institutionalize best practices. Regular refresher training and supportive supervision are key components of sustaining high levels of triage accuracy [14].

Limitations

This study has several limitations that should be acknowledged. First, it was conducted in a single ED, which may limit the generalizability of the findings to other institutions with different patient populations, staffing structures, or organizational cultures. Second, although the audit demonstrated significant short-term improvement, the follow-up period was relatively brief, and long-term sustainability of the observed gains was not formally evaluated. Third, the potential influence of the Hawthorne effect cannot be excluded, as triage staff were aware that performance was being audited, which may have temporarily influenced their behavior during the study period. Additionally, triage accuracy was assessed through guideline-based review rather than independent, blinded external validation, and formal inter-rater reliability between assessors was not evaluated, which may introduce the possibility of subjective interpretation when determining the expected ATS category. Moreover, the study focused on overall triage accuracy without separately analyzing patterns of under-triage and over-triage, which may provide further insight into patient safety implications. Future studies incorporating multicenter designs, longer follow-up periods, and more granular outcome analyses would strengthen the evidence base.

Finally, the improvement in ATS application after intervention in this audit supports the broader adoption of structured audit cycles as a means of aligning clinical practice with evidence-based guidelines. Such audits contribute not only to enhanced patient safety and departmental performance but also to organizational learning and culture transformation, reinforcing the central role of quality improvement in ED operations [15-17].

## Conclusions

This clinical audit demonstrated a significant and sustained improvement in the accuracy of ATS category assignment following the implementation of targeted educational and system-based interventions. Baseline triage performance was below the predefined standard; however, the reaudit cycle demonstrated that structured education, bedside coaching, and the use of cognitive aids were effective in improving adherence to ATS guidelines. These findings highlight the value of closed-loop clinical audit as a practical quality improvement tool and support the ongoing use of regular audit cycles to maintain high standards of triage practice and patient safety in the ED. Similar audit-based educational and system-level interventions may also be applicable in other EDs seeking to improve triage reliability and strengthen patient prioritization processes in busy clinical environments.
